# Age Distribution of Influenza Like Illness Cases during Post-Pandemic A(H3N2): Comparison with the Twelve Previous Seasons, in France

**DOI:** 10.1371/journal.pone.0065919

**Published:** 2013-06-05

**Authors:** Clément Turbelin, Cécile Souty, Camille Pelat, Thomas Hanslik, Marianne Sarazin, Thierry Blanchon, Alessandra Falchi

**Affiliations:** 1 INSERM, U 707, F-75012, Paris, France; 2 UPMC Univ Paris 06, UMR-S U707, F-75012, Paris, France; 3 INSERM, U 738, F-75018, Paris, France; 4 Université Paris Diderot, UMR-S 738, F-75018, Paris, France; 5 Université Versailles Saint Quentin en Yvelines, Versailles, France; 6 Assistance Publique Hôpitaux de Paris, Paris, France; 7 Service de Médecine Interne, Hôpital Ambroise Paré, Boulogne-Billancourt, France; 8 Laboratoire de Virologie, Université de Corse, Corte, France; National Institutes of Health, United States of America

## Abstract

In France, the 2011–2012 influenza epidemic was characterized by the circulation of antigenically drifted influenza A(H3N2) viruses and by an increased disease severity and mortality among the elderly, with respect to the A(H1N1)pdm09 pandemic and post-pandemic outbreaks. Whether the epidemiology of influenza in France differed between the 2011–2012 epidemic and the previous outbreaks is unclear. Here, we analyse the age distribution of influenza like illness (ILI) cases attended in general practice during the 2011–2012 epidemic, and compare it with that of the twelve previous epidemic seasons. Influenza like illness data were obtained through a nationwide surveillance system based on sentinel general practitioners. Vaccine effectiveness was also estimated. The estimated number of ILI cases attended in general practice during the 2011–2012 was lower than that of the past twelve epidemics. The age distribution was characteristic of previous A(H3N2)-dominated outbreaks: school-age children were relatively spared compared to epidemics (co-)dominated by A(H1N1) and/or B viruses (including the 2009 pandemic and post-pandemic outbreaks), while the proportion of adults over 30 year-old was higher. The estimated vaccine effectiveness (54%, 95% CI (48, 60)) was in the lower range for A(H3N2) epidemics. In conclusion, the age distribution of ILI cases attended in general practice seems to be not different between the A(H3N2) pre-pandemic and post-pandemic epidemics. Future researches including a more important number of ILI epidemics and confirmed virological data of influenza and other respiratory pathogens are necessary to confirm these results.

## Introduction

Infections with influenza viruses concern 10 to 20% of the worldwide population each year. Adults over 65 years-old (y), children under five and people suffering from particular medical conditions are the most at risk of complications, hospitalisations and deaths [1]. Influenza outbreaks dominated by A(H3N2) influenza viruses are associated with greater morbidity and mortality than A(H1N1), especially among the elderly [2], [3], [4], [5]. In humans, A(H3N2) viruses are considered to evolve faster than the A(H1N1) subtype [6]. Every three to eight years, predominant A(H3N2) viruses are replaced by a novel antigenic variant, prompting an update of the recommended influenza vaccine strain [7].

In France, as in other European countries, the 2011–2012 influenza outbreak, dominated by A(H3N2) viruses, was associated with higher frequency of severe outcomes among adults over 65 y, and higher mortality among those over 85, than during the last two seasons, which were dominated by pandemic A(H1N1)pdm09 viruses [8], [9]. Besides, more clusters of acute respiratory infection were notified during 2011–2012 in French nursing homes with respect to the 2003–2011 period [10]. It was hypothesized that the observed excess mortality among the elderly in 2011–2012 was related to the return of influenza A(H3N2) virus antigenically variant from the vaccine strain, potentially with added effects of a cold snap [9]. Indeed, a significant decrease of the trivalent influenza vaccine effectiveness against severe influenza cases in high-risk patients was reported this season [8]. However, the mortality was similar to that observed during the 2008–2009 outbreak dominated by A(H3N2) influenza viruses well-matched with the vaccine strain [9].

Whether the epidemiology of influenza this season differed from previous epidemics in France is unclear. Here, we analyse the age distribution of influenza like illness (ILI) cases reported by volunteer sentinel general practitioners (GPs) [11], in 2011–2012 and in the twelve previous outbreaks (pre-pandemic, pandemic 2009–2010, and post-pandemic 2010–2011). We also assess influenza vaccine effectiveness over this period.

## Methods

### Influenza data

The study was performed on the influenza epidemics from 1999–2000 to 2011–2012. Consultations for ILI in a general practice setting were the chosen morbidity indicator. These data were obtained from the French *Sentinelles* Network, a nationwide system based on voluntary and unpaid sentinel GPs who report weekly numbers of ILI consultations, age, sex, vaccinations status and some clinical characteristics of patients [12]. Influenza-like illness was defined as a sudden onset of fever over 39°C with myalgia and respiratory symptoms (cough, sore throat); no virological confirmation was performed at the individual level. The dominant circulating viral type(s) or subtype(s) during each epidemic was obtained from the World Health Organization’s (WHO) Flunet virological surveillance online database [13]: A(H1N1)pdm09, A(H1N1), A(H3N2), B or a combination. A significant number of type A viruses were not subtyped, so we adjusted each subtype count by reallocating the non-subtyped A count to each subtype according to the H1/H3 subtype ratio of the raw count given by the Flunet database (as described by Finkelman *et al*. [14]). The proportion was then calculated based on these corrected counts.

One subtype qualified as dominant if comprising more than 70% of the epidemic period’s influenza isolates except for the 2002–2003 outbreak. In France, during the 2002–2003 influenza epidemic when mostly type B was found to be circulating in general practice, A(H1N1) was mostly reported from hospital based tests. A type or subtype was considered “codominant” if it accounted for between 40 and 70% of the annual isolates [14], [15] (Table 1).

**Table 1:** Circulating viruses in France by epidemic (from FluNet database).

Epidemic Season	Proportion of swabs positive for types/subtypes from FluNet database^a^			Considered viral circulation
	A(H3N2)	A(H1N1)	B	
**1999–2000**	**99%**	<1%	<1%	A(H3N2)
**2000–2001**	9%	**70%**	21%	A(H1N1)
**2001–2002**	**82%**	<1%	18%	A(H3N2)
**2002–2003**	**9%**	**43%**	**48%**	B ^c^
**2003–2004**	**99%**	1%	<1%	A(H3N2)
**2004–2005**	**88%**	5%	7%	A(H3N2)
**2005–2006**	2%	**43%**	**55%**	B + A(H1N1)
**2006–2007**	**99%**	<1%	<1%	A(H3N2)
**2007–2008**	3%	**63%**	**34%**	A(H1N1) + B
**2008–2009**	**83%^b^**	3%^b^	15%^b^	A(H3N2)
**2009–2010**	<1%	**99%**	<1%	A(H1N1)pdm09
**2010–2011**	6%	**58%**	**36%**	A(H1N1)pdm09 + B
**2011–2012**	**92%**	5%	2%	A(H3N2)

a Proportion corrected to include not subtyped A viruses^ b^ Data for the 2008–2009 are from Grog network only.^ c^ A majority of type B was circulating in general practice. A(H1N1) was mostly reported from hospital based tests during the 2002–2003 season.

### Viral circulation

Of the 13 epidemics included in this study, the A(H3N2) subtype predominated in seven epidemics; the remaining six epidemics were dominated by A(H1N1) (N = 1), A(H1N1)pdm09 (N = 1), and B (N = 1); and co-dominated by A(H1N1) with B (N = 2) and A(H1N1)pdm09 with B (N = 1) (Table 1). The 2011–2012 influenza epidemic started in week 5 of 2012 (30^th^ January–5^th^ February), peaked in week 8 (20^th^–26^th^ February) and ended in week 12 (19^th^–25^th^ March), for a duration of eight weeks. This outbreak was dominated by the A(H3N2) subtype.

The protocol was conducted in agreement with the Helsinki declaration. We obtained authorization from the French Data Protection Agency (CNIL, registration number #471393).

### Influenza-like illness incidences rates

As previously reported [16], [17], ILI surveillance data has been shown to be a good proxy for influenza incidence in France and elsewhere.

Weekly regional ILI incidences were estimated by multiplying the mean number of reported cases per participating GP for a week by the total number of GPs in the area. National incidence was computed as a weighted sum of regional incidences (NUTS 2 level). Incidence rates (per 100,000 inhabitants) were obtained dividing incidences by yearly population size [18]. Volume activity of the population of *Sentinelles* GPs does not noticeably fluctuate over years as age distribution of their patients [19]. Age-specific incidence rates were estimated for the following age groups: 0 to 4 year-old (y), 5 to 17 y, 18 to 29 y, 30 to 44 y, 45 to 64 y, 65 to 74 y and 75 y or older.

We used a Poisson distribution to model the number of cases reported by *Sentinelles* GPs in regions and age-groups over each epidemic period. Variance of incidence was estimated using a normal-approximation allowing the computation of the 95% confidence intervals of the incidence rates. We then compared the incidence rates of the 2011–2012 epidemic to the average incidence rates of the last 10 pre-pandemic outbreaks (epidemics 1999–2000 to 2008–2009) using the mean and the variance of these two normal distributions.

### Determination of epidemic periods

Epidemic periods were determined by applying a periodic regression model including a linear trend, annual and semi-annual periodic terms on weekly ILI incidence rates below a cut-off value to estimate a baseline [20], [21]. Epidemics were then defined by at least two consecutive weekly incidence rates over the estimated baseline’s upper 90% prediction bound. For each influenza season (starting from September to the next year’s August), incidences were cumulated over all weeks included in the epidemic period as defined above. These epidemic periods will be referred as “season” in the following text. Each epidemic was named by the name of its influenza season (i.e. 2011–2012 epidemic, refer to the epidemic period occurred between September of 2011 to August of 2012).

### Relative illness ratio

The age-specific burden of illness was assessed with the relative illness ratio (RIR) [22]. This ratio divides the contribution of a specific age group *i* to ILI cases 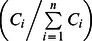 by its contribution to the general population 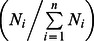:



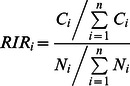



where *C_i_* was the number of ILI cases in an age group *i*, (there are *n* age groups in total) and *N_i_* was the total population in an age group *i*. *i* among ILI cases: a ratio above 1 indicates an excess risk. Besides, being standardized on epidemic size, it can be compared across epidemics. Confidence intervals (CI) were estimated with the exact Poisson method [23].

### Vaccine effectiveness

Vaccine effectiveness (VE) was estimated with the screening method using a “case-cohort” or “case-base” design [24]. Its principle is to calculate VE using the following equation:



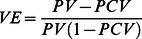



where PV is the proportion of vaccinated individuals in the general population and PCV is the proportion of vaccinated ILI cases [24]. In the simplified approach we used, proposed by Farrington *et al.* [25], the proportion of vaccinated individuals in the general population is assumed to be known (*i.e.* not estimated from a sample).

The PCV for each epidemic was estimated using *Sentinelles’* ILI cases including information on age and vaccine status at the individual level. Individuals with missing age or vaccination status were excluded. The proportion of vaccinated individuals in the general population (PV) was drawn from a phone-based post-epidemic survey on the influenza vaccine conducted yearly by a private independent organization (TNS/SOFRES) for a French Influenza Expert Group the *Groupement d’Etude et d’Information sur la Grippe* (GEIG) from a representative sample of the French population over 15 y. Vaccine effectiveness estimates were stratified using the following age strata in Farrington’s formula [25]: 15–64 y, over 65 years and overall using these two age strata. We did not adjust for potential confounding factors.In this study, we did not include VE for the 2009–2010 and 2010–2011 seasons, because the vaccination coverage was not available from GEIG.

## Results

### Incidence rates

**Figure 1:**
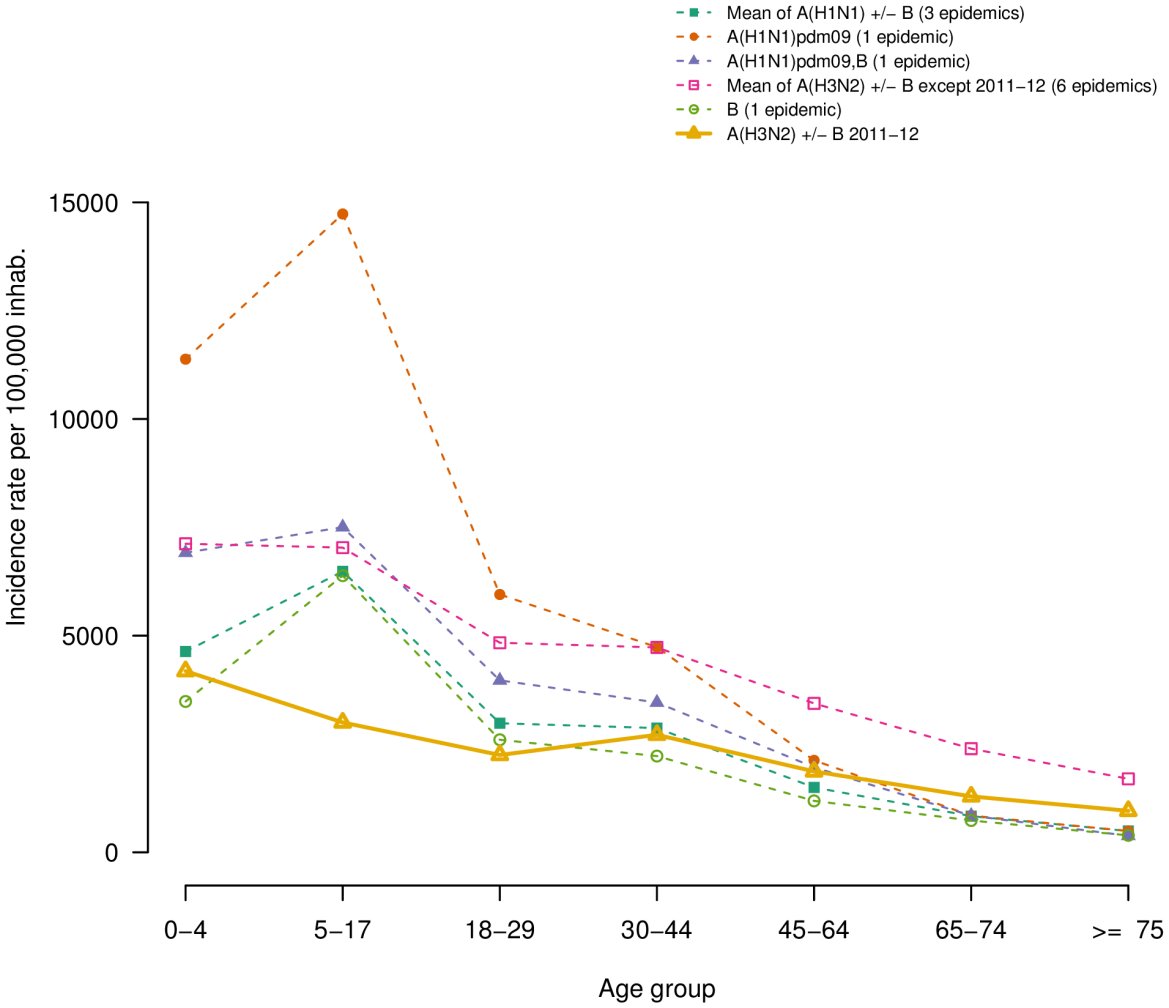
Age specific cumulated incidence rates for the 2011/2012 epidemic (solid line) by viral dominant or co-dominant subtypes for others epidemics from 1999–2000 epidemic (dashed lines). A(H1N1) and co-dominant A(H1N1),B before 2009 pandemic are grouped together). The number of epidemic included in each group is indicated between parentheses in the legend.

We report in Table 2, the cumulated incidence rates of ILI consultations of each epidemic and the corresponding 95% confidence interval, by age group and overall. The Figure 1 shows these age-specific ILI attack rates, grouping epidemics by according to the dominant virus type or subtype. As they had similar profiles, the pre-pandemic epidemics dominated by seasonal A(H1N1) viruses or co-dominated by seasonal A(H1N1) and B viruses were grouped together.

**Table 2:** Cumulated incidence rates and corresponding 95% confidence intervals of influenza-like illness per 100,000 inhabitants by age-group for epidemic seasons between 1999 and 2011.

	Age groups (years)							
Epidemics	0–4	5–17	18–29	30–44	45–64	65–74	> = 75	Overall
1999-00	5,629	5,401	5,874	6,215	5,947	4,805	3,292	5,592
	(5,117; 6,141)	(5,098; 5,705)	(5,552; 6,197)	(5,930; 6,500)	(5,675; 6,219)	(4,408; 5,201)	(2,927; 3,657)	(5,272; 5,913)
2000-01	4,149	6,060	2,883	2,435	1,170	727	493	2,626
	(3,611; 4,688)	(5,668; 6,453)	(2,610; 3,157)	(2,217; 2,653)	(1,028; 1,312)	(539; 915)	(330; 657)	(2,374; 2,877)
2001-02	7,096	6,050	3,808	4,289	2,813	1,829	1,221	3,881
	(6,355; 7,836)	(5,633; 6,466)	(3,464; 4,153)	(3,976; 4,602)	(2,571; 3,054)	(1,513; 2,144)	(935; 1,507)	(3,539; 4,224)
2002-03	3,481	6,384	2,597	2,221	1,187	734	391	2,521
	(2,916; 4,046)	(5,906; 6,862)	(2,287; 2,907)	(1,981; 2,460)	(1,021; 1,353)	(520; 949)	(229; 553)	(2,238; 2,805)
2003-04	10,654	9,110	5,481	3,971	2,253	1,542	1,487	4,645
	(9,552; 11,755)	(8,537; 9,682)	(4,968; 5,995)	(3,583; 4,360)	(1,975; 2,530)	(1,190; 1,895)	(936; 2,038)	(4,181; 5,110)
2004-05	6,217	8,034	5,259	5,423	4,030	2,894	2,444	5,072
	(5,645; 6,790)	(7,631; 8,438)	(4,925; 5,593)	(5,135; 5,711)	(3,799; 4,260)	(2,558; 3,230)	(2,131; 2,758)	(4,749; 5,396)
2005-06	4,432	7,280	2,423	1,961	1,067	728	367	2,583
	(3,947; 4,917)	(6,898; 7,662)	(2,196; 2,649)	(1,788; 2,134)	(950; 1,185)	(559; 898)	(248; 486)	(2,367; 2,798)
2006-07	5,236	6,355	3,642	3,617	2,155	1,468	793	3,375
	(4,659; 5,813)	(5,965; 6,745)	(3,336; 3,949)	(3,355; 3,879)	(1,970; 2,341)	(1,192; 1,743)	(597; 989)	(3,091; 3,660)
2007-08	5,318	6,110	3,630	4,206	2,255	1,048	610	3,426
	(4,806; 5,830)	(5,769; 6,450)	(3,363; 3,896)	(3,959; 4,453)	(2,093; 2,417)	(849; 1,246)	(464; 756)	(3,179; 3,673)
2008-09	7,893	7,239	4,951	4,851	3,422	1,815	937	4,478
	(7,033; 8,752)	(6,728; 7,750)	(4,429; 5,474)	(4,445; 5,258)	(3,112; 3,732)	(1,350; 2,281)	(671; 1,202)	(4,042; 4,915)
2009-10	11,381	14,732	5,950	4,734	2,122	847	491	5,549
	(10,678; 12,083)	(14,240; 15,223)	(5,624; 6,276)	(4,481; 4,988)	(1,971; 2,272)	(674; 1,020)	(367; 616)	(5,263; 5,834)
2010-11	6,914	7,504	3,968	3,457	1,962	845	381	3,514
	(6,420; 7,408)	(7,184; 7,824)	(3,725; 4,211)	(3,260; 3,655)	(1,831; 2,092)	(691; 999)	(282; 480)	(3,302; 3,727)
2011-12	4,182	2,997	2,245	2,711	1,867	1,290	957	2,279
	(3,757; 4,607)	(2,771; 3,222)	(2,045; 2,446)	(2,519; 2,903)	(1,729; 2,004)	(1,085; 1,495)	(788; 1,125)	(2,082; 2,476)

The 2011–2012 epidemic had overall a lower ILI attack rate than all twelve previous outbreaks (Table 2). The attack rate was highest among children under 5 y, but even in this age group, it remained in the lower range of all previous epidemics. The ILI attack rates for the 5–17 y and 18–29 y age groups were the lowest reported since 1999 and the third lowest for the 0–4 y age group (Figure 1 and Table 2). The ILI attack rates for 65–74 y and over 75 y age groups were higher than those reported during the pandemic and post-pandemic outbreaks but smaller to that of the A(H3N2)-dominated pre-pandemic outbreaks. Differences between ILI attack rates for 2011–2012 epidemic and average of 10 pre-pandemic seasons were significant for all age groups (p-value <0.0001; for over 75 y, p-value  = 0.01).

### Relative illness ratio

**Figure 2:**
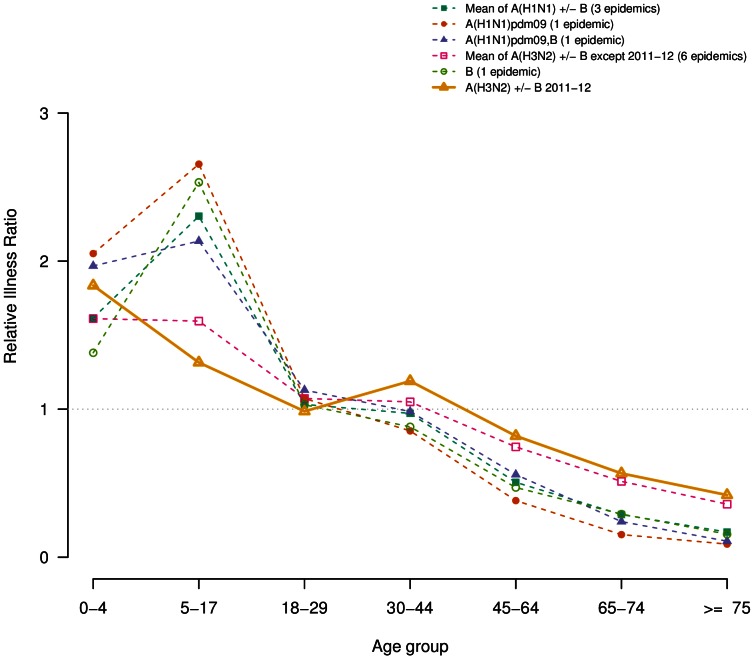
Relative Illness Ratio by age-group for the 2011/2012 epidemic (solid line) and mean of cumulated age-specific incidence by viral dominant or co-dominant subtypes for others epidemics from 1999–2000 epidemic (dashed lines). A(H1N1) and co-dominant A(H1N1),B before 2009 pandemic are grouped together). The number of epidemic included in each group is indicated between parentheses in the legend.

The mean RIR of each age group, by dominant virus subtype, are shown in Figure 2. Consistently across all epidemics, RIRs were highest in the young (<18 y) and decreased in adults (Table 3). Interestingly, in ILI epidemics not dominated by A(H3N2), school-age children had higher RIR than children under five. However, large confidence intervals do not allow further interpretation. Also, in the 2011–2012 epidemic, the RIR in adult did not decrease monotonically with age: the 30–44 y consulted more than the 18–29; yet again, confidence intervals are large. In 2011–2012 as in the other A(H3N2) epidemics, compared to A(H1N1) and/or B-dominated epidemics, the RIR profile had smaller variation around the unit, *i.e.* was slightly higher for younger age groups and lower for adults over 44 y. The lowest RIR in people over 65 y was observed during the 2009 A(H1N1) pandemic.

**Table 3:** Relative illness ratios and corresponding 95% confidence intervals of influenza-like illness by age-group for epidemic seasons between 1999 and 2011.

Epidemics	Age groups (years)						
	0–4	5–17	18–29	30–44	45–64	65–74	> = 75
1999-00	1.01	0.97	1.05	1.11	1.06	0.86	0.59
	(0.37; 2.17)	(0.55; 1.56)	(0.61; 1.68)	(0.71; 1.65)	(0.68; 1.58)	(0.36; 1.72)	(0.17; 1.47)
2000-01	1.58	2.31	1.1	0.93	0.45	0.28	0.19
	(0.75; 2.93)	(1.64; 3.16)	(0.65; 1.75)	(0.57; 1.43)	(0.22; 0.81)	(0.04; 0.9)	(0.01; 0.85)
2001-02	1.83	1.56	0.98	1.1	0.72	0.47	0.31
	(0.92; 3.24)	(1.01; 2.29)	(0.55; 1.61)	(0.71; 1.64)	(0.42; 1.16)	(0.13; 1.19)	(0.05; 1.03)
2002-03	1.38	2.53	1.03	0.88	0.47	0.29	0.16
	(0.62; 2.66)	(1.82; 3.43)	(0.59; 1.68)	(0.53; 1.38)	(0.24; 0.84)	(0.05; 0.93)	(0; 0.76)
2003-04	2.29	1.96	1.18	0.85	0.48	0.33	0.32
	(1.26; 3.83)	(1.34; 2.77)	(0.7; 1.86)	(0.51; 1.34)	(0.25; 0.85)	(0.06; 0.99)	(0.05; 1.03)
2004-05	1.23	1.58	1.04	1.07	0.79	0.57	0.48
	(0.51; 2.46)	(1.03; 2.33)	(0.59; 1.69)	(0.68; 1.61)	(0.48; 1.23)	(0.18; 1.35)	(0.13; 1.25)
2005-06	1.72	2.82	0.94	0.76	0.41	0.28	0.14
	(0.84; 3.11)	(2.06; 3.77)	(0.52; 1.57)	(0.43; 1.23)	(0.2; 0.75)	(0.04; 0.94)	(0; 0.71)
2006-07	1.55	1.88	1.08	1.07	0.64	0.43	0.23
	(0.73; 2.9)	(1.27; 2.69)	(0.62; 1.74)	(0.67; 1.62)	(0.37; 1.03)	(0.1; 1.18)	(0.03; 0.85)
2007-08	1.55	1.78	1.06	1.23	0.66	0.31	0.18
	(0.73; 2.89)	(1.19; 2.57)	(0.61; 1.72)	(0.8; 1.81)	(0.38; 1.06)	(0.05; 0.99)	(0.01; 0.75)
2008-09	1.76	1.62	1.11	1.08	0.76	0.41	0.21
	(0.88; 3.16)	(1.05; 2.38)	(0.64; 1.77)	(0.68; 1.64)	(0.46; 1.18)	(0.09; 1.15)	(0.02; 0.79)
2009-10	2.05	2.65	1.07	0.85	0.38	0.15	0.09
	(1.08; 3.53)	(1.91; 3.59)	(0.62; 1.73)	(0.5; 1.36)	(0.18; 0.71)	(0; 0.75)	(0; 0.59)
2010-11	1.97	2.14	1.13	0.98	0.56	0.24	0.11
	(1.02; 3.42)	(1.47; 2.99)	(0.66; 1.8)	(0.6; 1.53)	(0.31; 0.93)	(0.02; 0.89)	(0; 0.62)
2011-12	1.84	1.31	0.99	1.19	0.82	0.57	0.42
	(0.93; 3.26)	(0.81; 2.02)	(0.55; 1.64)	(0.76; 1.78)	(0.51; 1.24)	(0.17; 1.36)	(0.11; 1.1)

### Vaccine effectiveness

**Figure 3:**
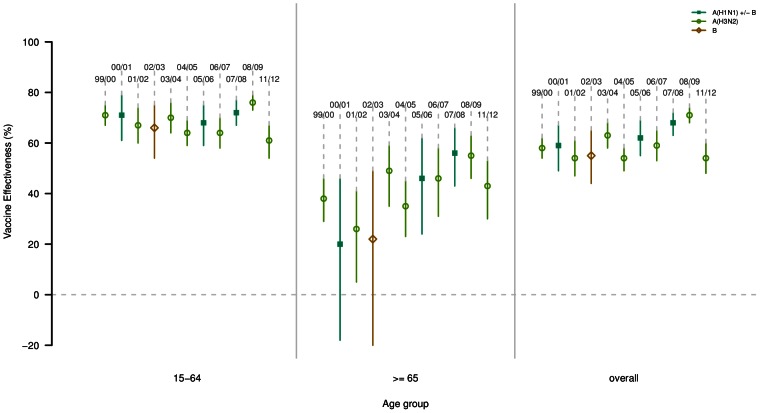
Effectiveness of trivalent seasonal influenza vaccine, for each epidemic, by age-group (15–64 year-old, over 65 year-old and overall) estimated by the French *Sentinelles* Network. Colors and types of point indicate the dominant or co-dominant viral subtype for each epidemic. Segments delimitate the 95% confidence intervals of the point estimates (circles or squared dots). Epidemic seasons are indicated above each estimation. Epidemics 2009–2010 and 2010–2011 are not shown.

The vaccine effectiveness, estimated for each outbreak except 2009–2010 and 2010–2011, is presented in Table 4 and Figure 3, for people 15–64 y and over 65 y, and overall. When considering all age groups, the VE estimated during the 2011–2012 outbreak (54%, 95% CI (48; 60)) were close to the lowest values for previous A(H3N2) epidemics. In the elderly, it was similar to that of previous A(H3N2) epidemics: 43%, 95% CI (30; 53).

**Table 4:** Vaccine effectiveness of people of 15–64 y and 65 and over, of the epidemics between 1999 and 2011 (in percent) and mismatch between dominant or co-dominant circulating strains and vaccine strains.

Epidemic ^a^	Vaccine effectiveness estimate and its 95% Confidence Interval (%)			Mismatch with vaccine strain ^b^
	15–64 y	> = 65 y	Overall	
**1999–00**	58 (54; 62)	38 (29; 46)	58 (54; 62)	–
**2000–01**	71 (61; 79)	59 (49; 67)	55 (49; 67)	–
**2001–02**	67 (60; 74)	26 ( 5; 41)	54 (47; 61)	–
**2002–03**	66 (54; 75)	22 (–20; 49)	55 (44; 65)	B
**2003–04**	70 (64; 76)	49 (35; 59)	63 (58; 68)	A(H3N2)
**2004–05**	64 (59; 69)	35 (23; 45)	54 (49; 58)	A(H3N2)^c^
**2005–06**	68 (59; 75)	46 (24; 62)	62 (55; 69)	B
**2006–07**	64 (58; 70)	46 (31; 58)	55 (53; 65)	–
**2007–08**	72 (67; 77)	56 (43; 66)	68 (63; 72)	B and A(H1N1)
**2008–09**	76 (73; 79)	55 (46; 63)	71 (68; 74)	–
**2011–12**	61 (54; 67)	43 (30; 53)	54 (48; 60)	A(H3N2)

^a^ Because vaccine effectiveness (VE) for 2009–2010 and 2010–2011 epidemics were estimated using different vaccination coverage sources (administrative source [44] and from GPs practices respectively) and strata (week for 2009–2010 and risk-group for influenza for 2010–2011 epidemic) we did not shown these VE estimated values in this study. ^b^ Indicate the viral dominant type or subtype when it differs from the vaccine strain for the season. Only mismatch with dominant type is considered. ‘–‘ indicate the circulating strains were close to the vaccine’s ones. ^c^ One of the 2 circulating A(H3N2) strains differed from the vaccine one.

## Discussion

This study compares the burden and age distribution of the 2011–2012 influenza outbreak with that of twelve previous ones, by applying a same methodology over a single and continued source of surveillance data: ILI cases reported by the *Sentinelles* GPs. We showed that the 2011–2012 was the mildest influenza outbreak observed since 1999 and that ILI consultation rates did not peak in school-age children (5–17 y), as usually observed in the previous epidemics. The relative risk of ILI showed variability by age and influenza subtype. As previously reported [22], the 2009 A(H1N1) pandemic displayed in our data high ILI attack rates in young children, which decreased with age. In our analysis, the lowest RIR was observed in the elderly during the circulation of A(H1N1)pdm09 influenza viruses. This difference was explained by the development of a lasting immunity against A(H1N1) viruses due to exposure to previous epidemics in elderly [26], [27].

In the 2002–2003 season, the only one dominated by influenza B viruses, the risk of ILI peaked in school-age children and decreased with age in agreement with some earlier studies in temperate countries in which influenza B risk peaked in preschool or school-age children [28], [29]. The same trend is observed for every year characterised by the circulation or co-circulation of A(H1N1) and/or B influenza viruses. These observations, are consistent with previous findings that lasting immunity to A(H1N1) influenza virus, possibly carried over from exposure to previous epidemics and pandemics, exists in the older population and decreases their risk of developing acute symptoms [26], [30].

During the six pre-pandemic ILI epidemics dominated by A(H3N2), the risk of ILI were highest in preschool and school-age children, with a risk in older adults more important with respect to those observed in the pandemic and the post-pandemic seasons (2009–2011). In the A(H3N2) post-pandemic outbreak the risk of ILI by age group was similar than those reported during the previous A(H3N2) pre-pandemic seasons here analysed. These data are in agreement with earlier studies reporting that adults experience higher rates of infection and reinfection with A(H3N2) than with other influenza types/subtypes [3], [31], [32].

It is known that A(H3N2) more commonly causes clinical illness in adults in the community and in institutional care compared with other influenza viruses [33]. During the 2011–2012 epidemic in France, 14-fold more clusters of acute respiratory infection were notified in nursing homes than the average annual number observed during the period 2003–2011 [10]. The duration of episodes, the attack rate and the case fatality among residents were unchanged [10]. In contrast, our analysis, based on consultations for ILI in the community show a similar risk of ILI among the elderly than during the last A(H3N2) epidemics since 1999. These discordant results could be partially explained by better outbreak reporting in French nursing homes and by the fact that the population of 65 y consulting in general practices usually have better health than the senior population residing in nursing homes. Another reason could be that our ILI definition underestimates the true influenza burden amongst elderly, especially vaccinated subjects, who usually develop atypical symptomatology (*e.g.* low fever). In fact, monitoring consultations for ILI as a proxy for influenza transmission in the community is a practical but heavily biased system. There is a risk of underestimation associated with those infected who do not seek medical care, as well as over-estimation associated with ILI cases caused by other pathogens than influenza [34].

In France, the new A(H3N2) variant, distinct from the vaccinal strain (A/Perth/16/2009) was reported for 31% of the total detected A(H3N2) viruses during the 2011–2012 outbreak. This proportion remained stable during the entire epidemic [10]. Even if the relationship between antigenic drift and clinical vaccine effectiveness is not well understood, it is known that a greater degree of antigenic mismatch may contribute to reducing vaccine effectiveness [35]. We estimated that the effectiveness of 2011–2012 influenza vaccines in preventing ILI this epidemic in people over 15 y was among the lowest measured in previous A(H3N2) epidemics. Preliminary estimates from the I-MOVE European study [36] suggested that, among the target groups for vaccination, the effectiveness of the 2011–2012 influenza vaccine was low to moderate against medically-attended ILI virologically confirmed as influenza A(H3N2). In a recent study in France, the effectiveness of the 2011–2012 trivalent influenza against severe virologically confirmed influenza cases in high-risk patients was significantly lower than the one of the 2010–2011 epidemic: 30%, 95% CI (22; 39) *versus* 53% (40; 67), respectively [8].

This study has several limitations. First, we studied ILI and not confirmed influenza cases, thus our results should be interpreted in the context of febrile symptomatic infections associated with respiratory symptoms and myalgia leading to a medical visit. However, respiratory pathogens other than influenza viruses might cause ILI, resulting in moderate positive predictive values of not virologically confirmed ILI [37]. In particular along with influenza A and B viruses, adenoviruses (AdV), respiratory syncytial virus (RSV), enteroviruses (EVs), human rhinovirus(HRV), and parainfluenza viruses (PiVs) are regarded as important pathogens with the potential to cause ILI. As previously reported [38] influenza viruses and RSV accounted for at least 50% of respiratory viruses identified in ILI patients, and thus there is a substantial potential for confusion between illness caused by influenza and those caused by RSV even if influenza accounting for a much greater proportion of confirmed viruses during the ILI peak weeks, especially in adult age groups. To minimize confusion, in the present study all data were computed (incidences rates, relative illness ratio) over the epidemic period for each influenza season. Overall, even if all-ages ILI rates could be a good proxy for influenza virology data, there is no evidence for age specific data, thus results here shown have to be interpreted taking into account this limit. Moreover, the use of ILI, a non-specific influenza outcome, as a primary endpoint for estimating the effectiveness of influenza vaccines can bias vaccine effectiveness estimates downward.

Second, we focused on the last thirteen epidemics, since the 1999–2000 epidemics, as vaccination and prevention behaviours are susceptible to change over a long period of time. In France, until 1999–2000 the influenza vaccine was free of charge only for people over 70, but since 2000–2001, people between 65 and 69 were also eligible for free vaccinations.

Thirdly, biases due to differential healthcare-seeking behaviour in patients have not been measured when estimating vaccine effectiveness, in particular healthy user bias in the senior population, whereby elderly patients with poorer prognosis may be less likely to receive a vaccine compared to healthy seniors [39], [40]. On the other hand, individuals at higher risk for influenza are more likely to be vaccinated than individuals at lower risk. Thus, the different characteristics between groups may lead to erroneous estimation of the vaccine effectiveness.

Moreover, the ILIs incidences rates have been highly influenced by the health seeking behavior, during the spread of the 2009 pandemic. In early September 2009, while reports of ILI were increasing in medical practice-based surveillance in France and other European countries, the detection of pandemic influenza virus remained sporadic [41]. This finding was attributed to the circulation of other respiratory viruses and, to an increased propensity of patients with ILI to seek medical advice due to increased anxiety in the pandemic context. This finding was also observed for ILI incidence rates reported by the French *Sentinelles* Network [42].

Our results could also be biased due to the fact that the two samples we used are not drawn from the same population: the GEIG sample is drawn from the general population whereas the *Sentinelles* sample is not. Nevertheless, as the *Sentinelles* network is an ongoing system, and the GEIG repeats the poll regularly, we can provide real-time estimates of the VE for each epidemic season. Considering these temporal series and assuming that bias is the same from 1 year to another, we can compare estimates of VE to those of the previous [43].

In conclusion, the age distribution of ILI cases attended in general practice seems to be not different between the A(H3N2) pre-pandemic and post-pandemic epidemics. Future researches including a more important number of ILI epidemics and confirmed virological data of influenza and other respiratory pathogens are necessary to confirm these results.
